# Tanshinone IIA reduces AQP4 expression and astrocyte swelling after OGD/R by inhibiting the HMGB1/RAGE/NF-κB/IL-6 pro-inflammatory axis

**DOI:** 10.1038/s41598-022-17491-7

**Published:** 2022-08-18

**Authors:** Zhaohua Tang, Gang Yang, Zhengbu Liao, Feilan Chen, Song Chen, Wentao Wang, Gang Huo, Xiaochuan Sun, Xiaoshu Wang

**Affiliations:** 1 Department of Neurosurgery, The First Affiliated Hospital of Chongqing Medical University, No. 1 Youyi Road, Chongqing, 400016 China; 2 Animal Experiment Center, Chongqing Medical University, No. 1 Yixueyuan Road, Chongqing, 400016 China; 3 Department of Neurosurgery, The Affiliated Hospital of Northwest University, No.10, East Section of Fengcheng Third Road, Xi’an, 710082 China

**Keywords:** Drug discovery, Medical research, Molecular medicine, Neurology, Neuroscience, Cellular neuroscience, Molecular neuroscience

## Abstract

This study aimed to investigate the role of tanshinone IIA (TSO IIA) in astrocytic swelling caused by ischemia–reperfusion-like injury in an in vitro model and the molecular mechanisms underlying this effect. Primary brain astrocytes were cultured under conditions of glucose and oxygen deprivation and reoxygenation (OGD/R). The study explored the effects of TSO IIA treatment on cell swelling and injury and the protein levels of aquaporin 4 (AQP4) in the plasma membrane. It then examined the involvement of the high-mobility group box protein 1 (HMGB1)/receptors for advanced-glycation end products (RAGE)/nuclear factor-kappa B (NF-κB)/interleukin-6 (IL-6) pro-inflammatory axis in TSO IIA-mediated protection. The treatment with TSO IIA alleviated OGD/R-induced astrocytic swelling and the overclustering of AQP4 protein in the plasma membrane. In addition, TSO IIA significantly reduced the overexpression of HMGB1 and the high levels of the NF-κB protein in the nucleus and of the IL-6 protein in the cytoplasm and extracellular media induced by OGD/R. The combination of TSO IIA and recombinant HMGB1 reversed these effects. The inhibition of the RAGE, the receptor of HMGB1, induced results similar to those of TSO IIA. In addition, exogenous IL-6 reversed TSO IIA-mediated effect on AQP4 overclustering and cell swelling. TSO IIA significantly reduced astrocyte swelling after OGD/R injury in vitro, via blocking the activation of the HMGB1/RAGE/NF-κB/IL-6 pro-inflammatory axis and thereby decreasing the expression of AQP4 in the plasma membrane.

## Introduction

Brain edema following stroke, trauma, tumor growth, or infection is closely associated with various severe negative consequences^1,2^. Astrocytes are the main glial cell type in the central nervous system (CNS). Their swelling is an important component of cerebral edema due to abnormal accumulation of intracellular fluid and is caused mainly by pro-inflammatory cytokines^3,4^. Current treatments for brain edema are limited to osmotic therapies and surgical decompression.

Tanshinone IIA (TSO IIA) is an important lipophilic diterpene extracted from *Salvia miltiorrhiza* Bunge, a herbal medicine with many active ingredients widely distributed in Japan and China^5^. Increasing evidence suggests that TSO IIA has a wide range of anti-inflammatory^6^, antiapoptotic^7^, antioxidative^8^, and antitumor activities^9^. TSO IIA and its derivatives have been widely used as prescription treatments for stroke and angina pectoris^10–13^. Recently, TSO IIA has been reported to reduce cerebral edema and provide protection from cerebral ischemia–reperfusion or traumatic injury in rats through its anti-inflammatory effects^14–16^. However, the specific molecular mechanisms underlying these effects remain unclear.

Treating astrocyte swelling is important for avoiding the damaging effects of brain edema^17^. Aquaporin 4 (AQP4), a major member of the aquaporin family in the CNS, is highly expressed in astrocytes and plays an integral role in developing cytotoxic edema^18–20^.

Inflammation is mainly caused by the secretion of injury-related molecules by local cells (such as microglia and astrocytes) in CNS diseases^17,21^. Increasing evidence suggests that neuroinflammation is critical in the pathogenesis of brain edema^22,23^. High-mobility group box protein 1 (HMGB1) has been recently reported to be an important regulator in neuroinflammation^24,25^. It activates pattern-recognition receptors, such as receptors for advanced-glycation end products (RAGE), promotes the activation of the nuclear factor-kappa B (NF-κB), and triggers inflammatory responses in astrocytes, immune-competent cells, and neurons^25–28^. Furthermore, HMGB1 and its downstream pro-inflammatory cytokines such as interleukin-6 (IL-6) are associated with AQP4 expression and brain edema^29^.

This study investigated the effects of TSO IIA on astrocyte swelling after oxygen–glucose deprivation and reoxygenation (OGD/R) injury in vitro to identify the mechanisms underlying the effect of TSO and its potential in treating cerebral edema. Further, it examined whether these effects were related to regulating AQP4 expression in the plasma membrane through the HMGB1/RAGE/NF-κB/IL-6 pro-inflammatory axis.

## Results

### Tanshinone IIA reduced OGD/R-induced astrocytic swelling

**Figure 1:**
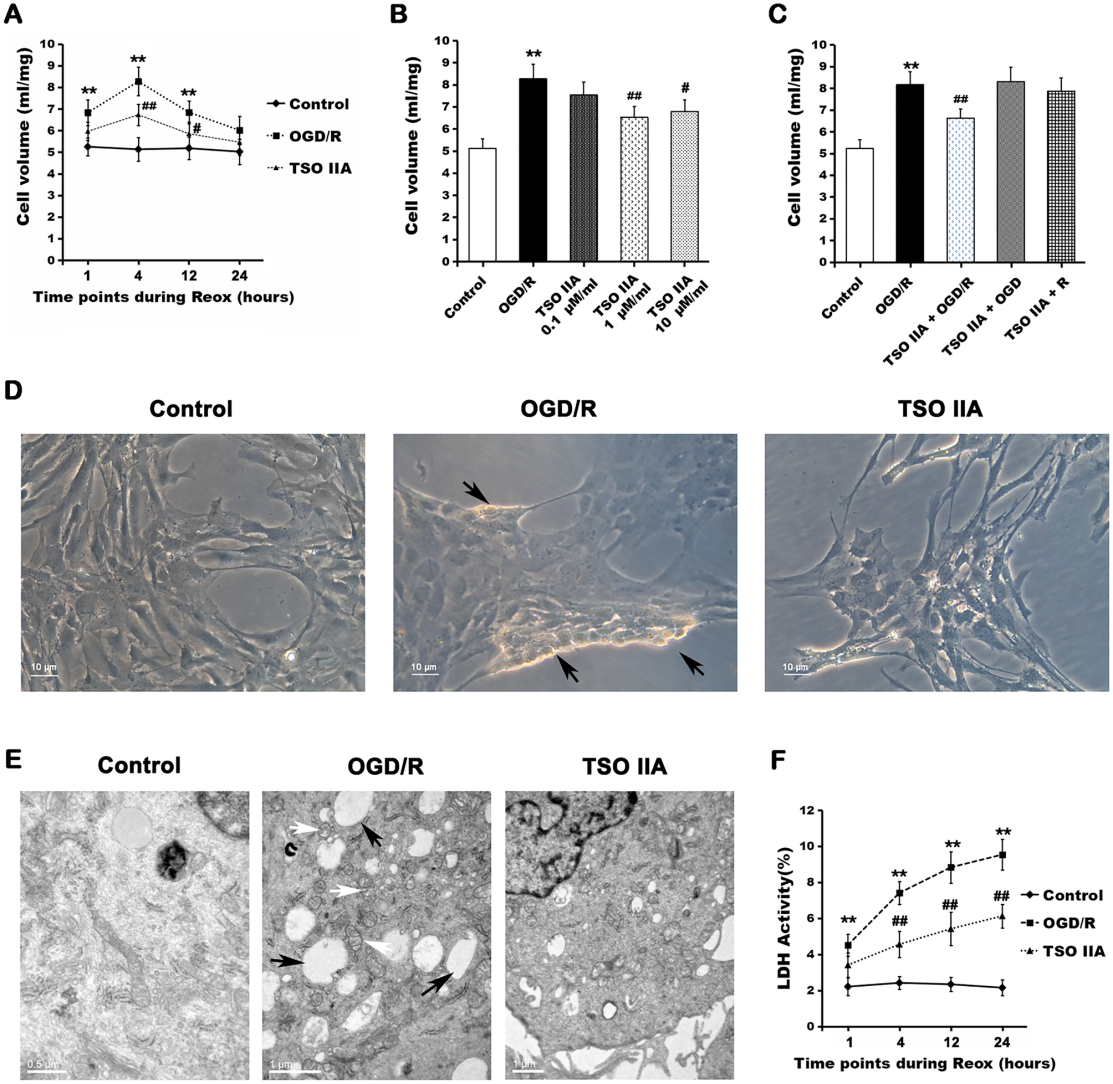
TSO IIA reduced astrocytic swelling and injury after OGD/R. (**A**) Cell volume quantification after OGD/R in untreated cells and cells treated with TSO IIA (1 μmol/mL) (*n* = 5 independent cultures). (**B**) Dose-dependent effect of TSO IIA on cell volume. 0.1, 1, or 10 μmol/mL of TSO IIA was added 24 h before and throughout the OGD/R process (*n* = 5 independent cultures). (**C**) Time-dependent effect of TSO IIA on astrocytic swelling. TSO IIA (1 μmol/mL) was added 24 h before and throughout the OGD/R (TSO IIA + OGD/R) process, only during the OGD phase (TSO IIA + OGD), or only during the reoxygenation phase (TSO IIA + R) (*n* = 5 independent cultures). (**D**) Phase-contrast imaging showing cell morphological changes under different treatments. Astrocytic swelling is depicted by black arrows (*n* = 5 independent cultures). (**E**) Stereoscan photographs showing an increase in lysosome number (black arrows) and mitochondria swelling (white arrows) (*n* = 5 independent cultures). (**F**) LDH activity analysis showing the protective effect of TSO IIA on OGD/R injury (*n* = 5 independent cultures) (The data in cell volume and LDH activity test are expressed as mean ± SEM. One-way ANOVA resulted in *P* < 0.01, *P < 0.05 versus the control; **P < 0.01 versus the control; ^#^*P* < 0.05 versus OGD/R; ^##^*P* < 0.01 versus OGD/R).

The changes in cell volume 1, 4, 12, and 24 h after OGD/R under different treatment conditions were measured to determine whether TSO IIA had a protective effect on astrocyte swelling. Following OGD/R treatment, the cell volume increased significantly and reached a peak after 4 h of reoxygenation (8.27 ± 0.66 mL/mg in the OGD/R group vs 5.13 ± 0.55 mL/mg in the control group; *P* < 0.01, Fig. 1A). Treatment with TSO IIA for 24 h prior to and throughout the OGD/R phase significantly reduced the increase in cell volume after 4 and 12 h of reoxygenation (both *P* < 0.05 vs OGD/R group). As shown in Fig. 1B, TSO IIA was found to be the most protective at the concentration of 1 μM/mL (*P* < 0.01 vs OGD/R group, Fig. 1B), with no statistically significant differences between the 1 μM/mL and 10 μM/mL concentrations (*P* > 0.01, Fig. 1B). However, the exposure of TSO IIA only in the OGD or reoxygenation phase did not have any effect on cell volume (both *P* > 0.05 vs OGD/R group, Fig. 1C). The control cultures treated with TSO IIA showed no changes in cell volume (data not shown).

The cell morphological and ultrastructural changes were inspected under light and electron microscopes to further confirm the anti-edema protective effect of TSO IIA. As shown in Fig. 1D, TSO IIA treatment significantly alleviated swelling and roundness, led to thickened and shortened processes and edge shrinkage, and significantly increased the refractive index caused by OGD/R. The ultrastructural changes characterized by mitochondrial swelling and increased lysosome number were remarkably reduced after TSO IIA treatment (Fig. 1E). Furthermore, a time-dependent increase in lactate dehydrogenase (LDH) activity was observed during the reoxygenation stage, which was also significantly attenuated by TSO IIA (*P* < 0.01 vs OGD/R group, Fig. 1F).

### TSO IIA reduced the clustering of AQP4 in the plasma membrane after OGD/R

**Figure 2:**
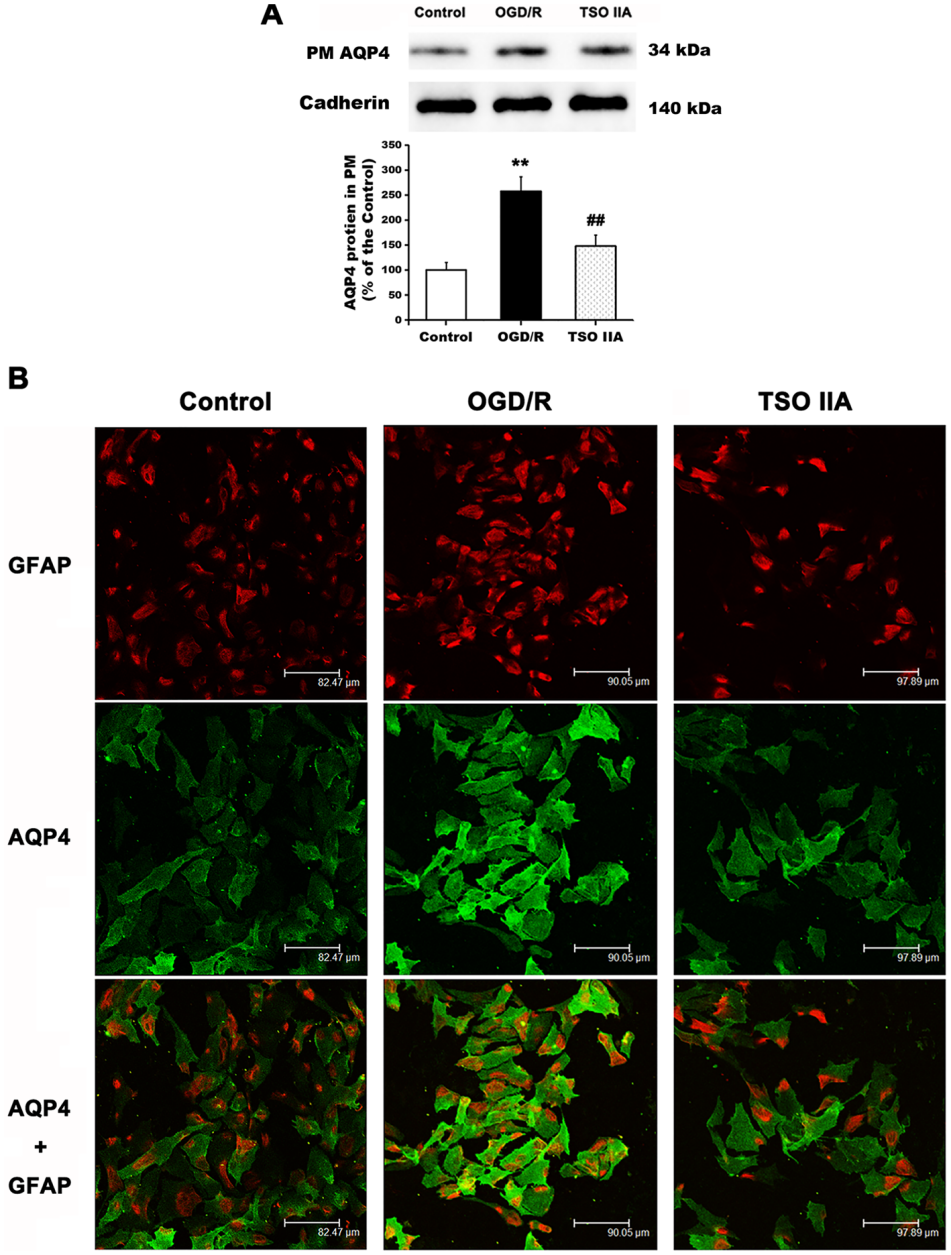
TSO IIA treatment decreased the clustering of AQP4 in the plasma membrane after OGD/R. (**A**) Western blot analysis showing the AQP4 protein in the plasma membrane after OGD/R in untreated cells or cells treated with TSO IIA. (The data are expressed as mean ± SEM, *n* = 5 independent cultures. One-way ANOVA resulted in *P* < 0.01, **P < 0.01 versus control; ^##^*P* < 0.01 versus OGD/R). (**B**) Double-labeling immunofluorescence staining confirmed AQP4 protein clustering in the plasma membrane under different treatment conditions (*n* = 5 independent cultures).

The expression of AQP4 protein was measured to investigate the effect of TSO IIA on AQP4 clustering in the plasma membrane during astrocytic swelling after OGD/R. Western blot results showed that AQP4 protein expression in the plasma membrane significantly increased by 161.3 ± 29.7% compared with the control group after 4 h of reoxygenation (*P* < 0.01 vs control group; Fig. 2A). The treatment with TSO IIA significantly relieved OGD/R-induced AQP4 increase in the plasma membrane (*P* < 0.01 vs OGD/R group). AQP4 clustering in the plasma membrane was then assessed using immunofluorescence after 4 h of reoxygenation. After OGD/R, the excessive aggregation of the AQP4 protein occurred in the plasma membrane of astrocytes, which was significantly reduced after TSO IIA treatment (Fig. 2B).

### TSO IIA inhibited HMGB1-induced inflammatory axis, AQP4 clustering in the plasma membrane, and cell swelling

**Figure 3:**
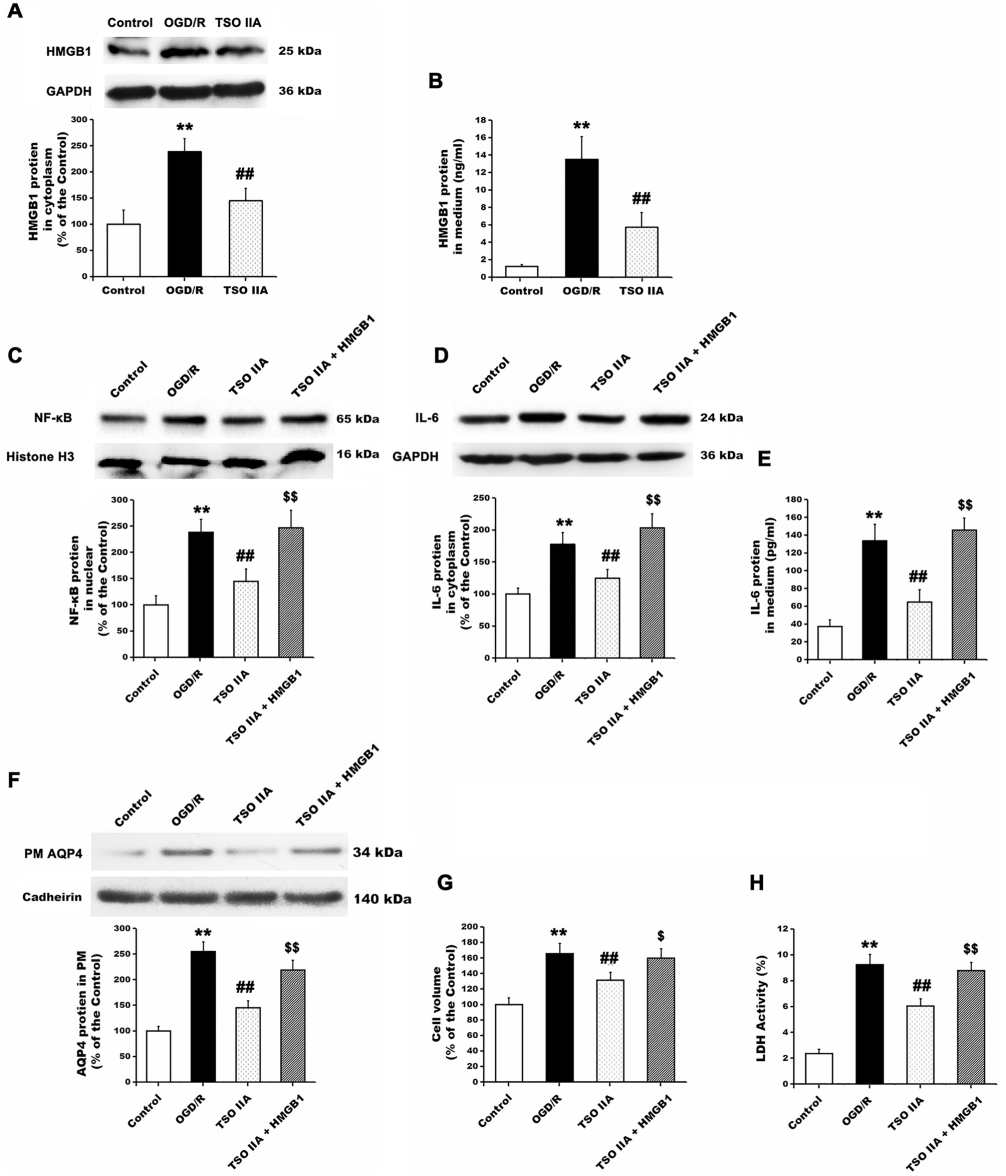
TSO IIA reduced cell swelling through inhibition of the HMGB1-activated inflammatory axis. (**A** and **B**) HMGB1 expression in the cytoplasm and culture medium after OGD/R, detected by Western blot and ELISA analysis in untreated cells and cells treated with TSO IIA (*n* = 5 independent cultures). (**C**) NF-κB protein accumulation in the nucleus in astrocytes in response to vehicle, TSO IIA, or both TSO IIA and rHMGB1 (*n* = 5 independent cultures). (**D** and **E**) IL-6 protein expression in the cytoplasm and medium after OGD/R in cells in response to vehicle, TSO IIA, or both TSO IIA and rHMGB1 (*n* = 5 independent cultures). (**F**–**H**) AQP4 clustering in the cytoplasm, cell volume, and LDH activity after OGD/R detected from cells in response to vehicle, TSO IIA, or both TSO IIA and rHMGB1 (*n* = 5 independent cultures). (The data of the aforementioned test are expressed as mean ± SEM. One-way ANOVA resulted in *P* < 0.01, **P < 0.01 versus control; ^##^*P* < 0.01 versus OGD/R; CM: plasma membrane).

The cells were first treated with or without TSO IIA and HMGB1 expression in the cytoplasm, and the surrounding medium was evaluated to determine whether the decrease in TSO IIA-mediated astrocyte swelling was due to the regulation of the HMGB1-activated inflammatory axis. The Western blot analysis showed that HMGB1 expression in the cytoplasm of astrocytes significantly increased by 138.5% ± 24.6% compared with the control cells after OGD/R (*P* < 0.01 vs control group, Fig. 3A). Further, the enzyme-linked immunosorbent assay (ELISA) showed that HMGB1 expression in the medium after OGD/R was increased by 375.2% ± 45.5% (*P* < 0.01 vs control group, Fig. 3B). TSO IIA treatment blocked the OGD/R-induced increase in the level of HMGB1 protein in the cytoplasm and medium to 144.6% ± 23.7% and 136% ± 49.6% of that in the control group, respectively (both *P* < 0.01 vs OGD/R group).

The cells were then treated with the vehicle, TSO IIA, or TSO IIA + rHMGB1, and the protein levels of NF-κB in the nucleus and those of IL-6 in the cytoplasm and extracellular medium after OGD/R were evaluated. As shown in Fig. 3, the protein levels of NF-κB in the nucleus and those of IL-6 in the cytoplasm or extracellular medium were significantly increased by 138.5% ± 24.6%, 77.5% ± 18.6%, and 258.9% ± 50.3%, respectively, after OGD/R (all *P* < 0.05 vs control group, Fig. 3C–E), while TSO treatment inhibited the increase in these protein levels (all *P* < 0.05 vs OGD/R group). Furthermore, the combined treatment of TSO IIA and rHMGB1 reversed the TSO IIA-mediated changes (all *P* < 0.05 vs TSO IIA group). TSO IIA and TSO IIA + rHMGB1 treatments were also found to play similar roles in regulating plasma membrane AQP4, cell volume, and LDH activity (all *P* < 0.05, TSO IIA + rHMGB1 group vs TSO IIA group, Fig. 3F–H).

### Role of RAGE in TSO IIA-mediated reduction of astrocyte swelling

**Figure 4:**
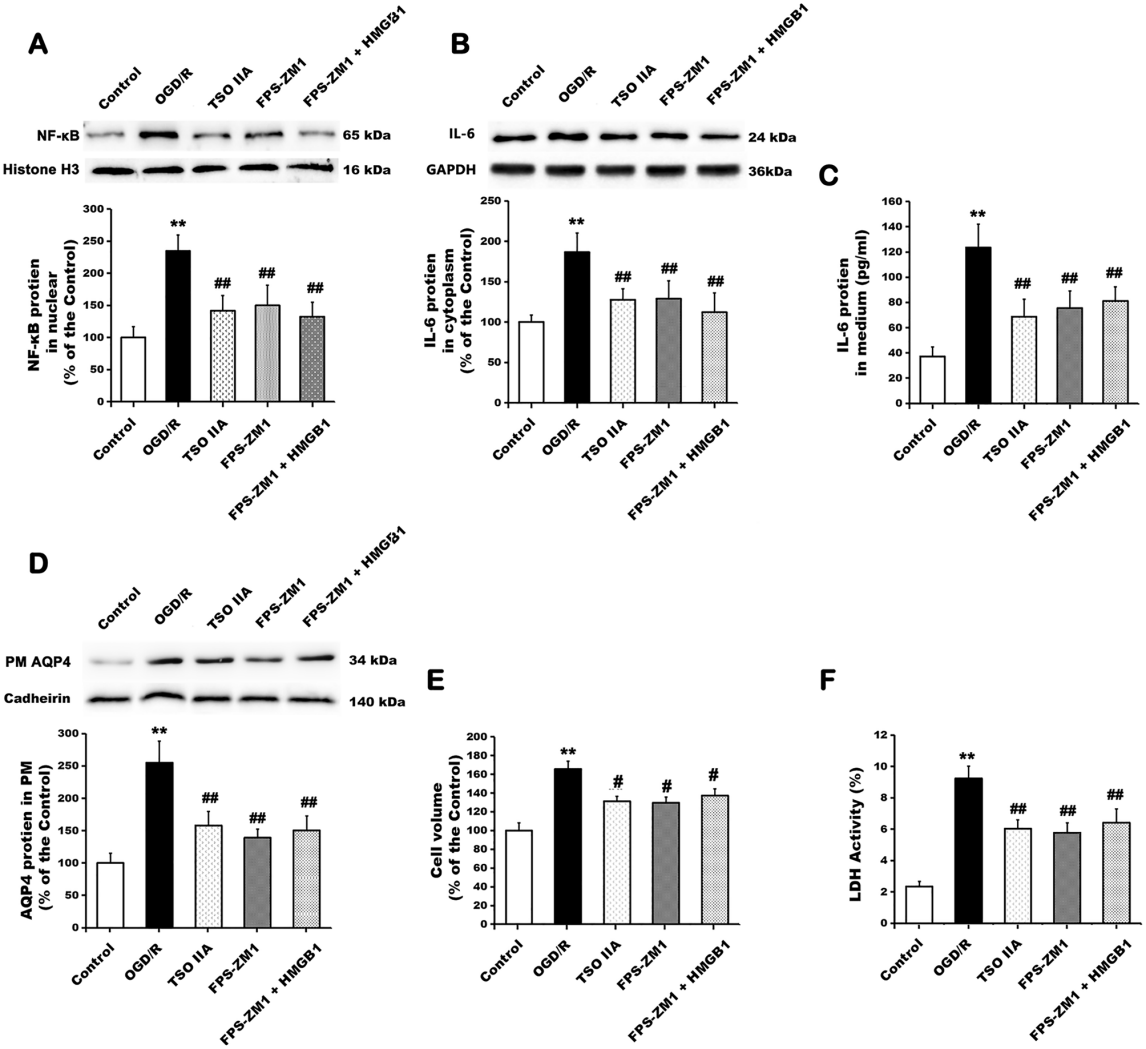
Effects of TSO IIA and FPS-ZM1 on the HMGB1-induced inflammatory axis, AQP4 clustering in the plasma membrane, and astrocytic swelling. (**A**–**C**) NF-κB protein accumulation in the nucleus and IL-6 protein expression in the cytoplasm and medium as examined by Western blot or ELISA after OGD/R in cells exposed to vehicle, TSO IIA, FPS-ZM1, or both FPS-ZM1 and HMGB1 (*n* = 3 independent cultures). (**D**–**F**) AQP4 protein clustering in the plasma membrane, cell volume, and LDH activity after OGD/R was detected in cells in response to vehicle, TSO IIA, FPS-ZM1, or both FPS-ZM1 and HMGB1 (*n* = 3 independent cultures). (The data of the aforementioned test are expressed as mean ± SEM. One-way ANOVA resulted in I < 0.01, **P < 0.01 versus control; ^##^*P* < 0.01 versus OGD/R; CM: plasma membrane).

The cells were treated with vehicle, TSO IIA, RAGE inhibitor FPS-ZM1, or both FPS-ZM1 and HMGB1 to clarify further the involvement of RAGE, which is one of the HMGB1 receptors in TSO IIA-mediated reduction of astrocyte swelling. The increases in the protein levels of NF-κB in the nucleus and those of IL-6 in the cytoplasm and medium after OGD/R were inhibited by FPS-ZM1 treatment (all *P* < 0.05 vs OGD/R group, Fig. 4A–C). These results were not significantly different from those after TSO IIA treatment (all *P* > 0.05). Furthermore, the combination of FPS-ZM1 and HMGB1 did not change the aforementioned effects mediated by FPS-ZM1 (all *P* > 0.05 vs FPS-ZM1 group) in terms of the regulation of AQP4 expression in the plasma membrane, cell volume, and LDH activity (Fig. 4D–F).

### TSO IIA reduced IL-6-mediated AQP4 expression and cell swelling

**Figure 5:**
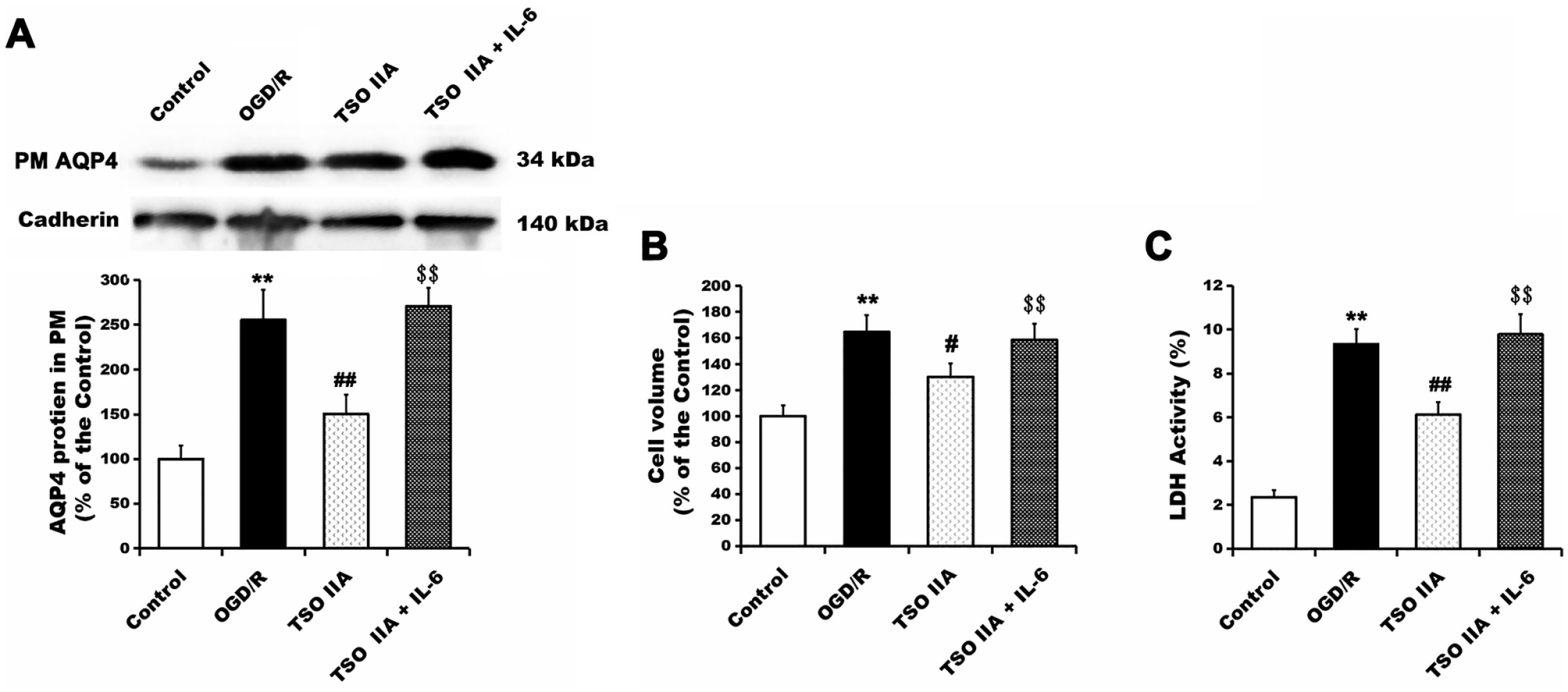
Role of IL-6 in TSO IIA-mediated inhibition of AQP4 overclustering in the cytoplasm and astrocyte swelling after OGD/R. (**A**) AQP4 expression in the plasma membrane after OGD/R in cells subjected to vehicle, TSO IIA, or both TSO IIA and IL-6 (*n* = 3 independent cultures). (**B** and **C**) Cell volume and LDH activity after OGD/R was detected in cells subjected to vehicle, TSO IIA, or both TSO IIA and IL-6 (*n* = 3 independent cultures). (The data of the aforementioned test are expressed as mean ± SEM. One-way ANOVA resulted in *P* < 0.01, **P < 0.01 represents versus control; ^##^*P* < 0.01 versus OGD/R; PM, plasma membrane).

The cells were treated with vehicle, TSO IIA, or TSO IIA and IL-6 to evaluate whether IL-6 was a key inflammatory factor in TSO IIA-mediated regulation of AQP4 expression in the plasma membrane and astrocyte swelling. Then, the protein levels of AQP4 in the plasma membrane, cell volume, and LDH activity were examined. Compared with TSO IIA-treated cells, the cells treated with TSO IIA + IL-6 displayed increased protein levels of AQP4 in the plasma membrane (all *P* < 0.05, Fig. 5A). The combination of TSO IIA and IL-6 reversed the decreases in cell volume and LDH activity induced by TSO IIA treatment alone (all *P* < 0.05 vs TSO IIA group, Fig. 5B,C).

## Discussion

**Figure 6:**
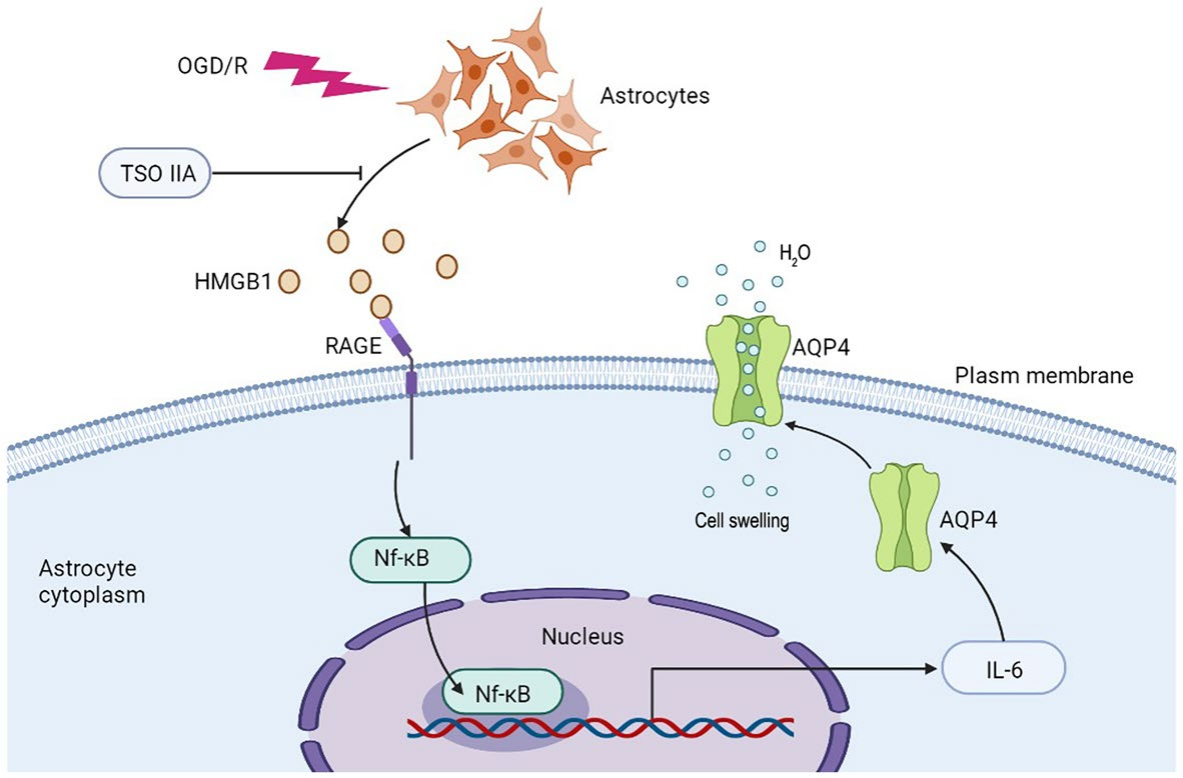
Schematic illustration of TSO IIA preventing AQP4 overexpression in PM and astrocyte swelling after OGD/R by inhibiting the HMGB1/RAGE/NF-κB/IL-6 pro-inflammatory axis.

The therapeutic options to prevent brain edema in CNS diseases are extremely limited, and hence neuroprotective therapies are urgently needed. The present study explored the effects of TSO IIA treatment to show for the first time that (1) TSO IIA protected against astrocyte swelling after OGD/R injury possibly by suppressing AQP4 expression in the plasma membrane and (2) by blocking the activation of the HMGB1/RAGE/NF-κB/IL-6 pro-inflammatory axis (Fig. 6).

TSO IIA is a lipophilic diterpene extracted from *Salvia miltiorrhiza* Bunge^30^. TSO IIA has been shown to prevent or slow the progression of various diseases, including CNS, cardiovascular, and cerebrovascular diseases due to its anti-inflammatory and anti-atherosclerotic properties^11^. Huang et al. found using a traumatic brain injury rat model that TSO IIA treatment downregulated the expression levels of inflammatory factors and AQP4, and decreased brain edema^15^. In a transient middle cerebral artery occlusion and reperfusion model, also in rats, Song et al. found that TSO IIA could significantly reduce neuroinflammation and infarct volume, improve neurological deficits, and reduce brain edema^14^. However, the specific molecular mechanisms underlying the effect of TSO IIA in alleviating brain edema remain unclear. In the present study, using astrocytes in culture, treatment with TSO IIA significantly inhibited the OGD/R injury-induced increase in the astrocyte volume. This protective effect in swelling was also consistent with the changes in cell morphology, ultrastructure, and cytotoxicity. These results confirmed the protective role of TSO IIA in edema in an in vitro model of ischemia and reperfusion-like injury.

In addition, the results of this study showed the upregulation of AQP4 in the plasma membrane and its association with astrocytic swelling following OGD/R. Specifically, the treatment with TSO IIA resulted in a significant reduction of AQP4 overclustering in the plasma membrane of astrocytes. AQP4 is the main water channel protein found in astrocytes and plays a role in facilitating transmembrane water movement in the CNS^31^. Furthermore, AQP4 was identified as a major player in astrocyte swelling and brain edema in various CNS diseases^31^. Under pathological conditions, the overexpression of AQP4 in the plasma membrane leads to inappropriate cellular uptake of water in astrocytes, resulting in brain edema^32^. Prior in vitro and in vivo studies reported that blocking the increase in the membrane AQP4 protein in astrocytes could effectively prevent cellular swelling or brain edema^33^. Thus, the results of this study suggested that TSO IIA-mediated abrogation of AQP4 overexpression in the plasma membrane was important for protecting astrocytes from swelling following OGD/R.

The putative regulatory mechanisms underlying the effect of TSO IIA treatment on AQP4 aggregation in the plasma membrane and astrocyte swelling need further exploration. In previous studies, extracellular HMGB1 overexpression activated downstream signaling pathways and enhanced the expression of inflammatory cytokines, such as IL-6, TNF-α, and IL-1β^34,35^. In the brain, HMGB1 and associated downstream inflammatory signaling pathways have been linked to significantly increased brain edema after traumatic brain injury, cerebral ischemia, and hepatic encephalopathy^36^. Importantly, the inhibition of HMGB1 led to decreased inflammation and improvement in brain edema^29,37,38^. The upregulation of HMGB1 after OGD/R injury in vitro has been shown to promote NF-κB activation and IL-6 secretion and lead to a decrease in astrocyte swelling^28,39^. In agreement with previous studies, the present study showed that the expression of intracellular and extracellular HMGB1 significantly increased following OGD/R and was accompanied by an increase in the nuclear transport of NF-κB protein and secretion of IL-6. The treatment of cells with TSO IIA abolished the OGD/R-induced changes. Therefore, the downregulation of HMGB1 expression, and consequently of its downstream inflammatory response, plays an important role in edema. Corroborating this finding, astrocytes were exposed to TSO IIA and exogenous HMGB1. As expected, the co-treatment reversed the effects of TSO IIA on the NF-κB activation, IL-6 secretion, AQP4 over-aggregation in the plasma membrane, and cell swelling, further suggesting that the target of TSO II-mediated neuroprotection was the pro-inflammatory factor HMGB1.

Secreted HMGB1 binds to its receptors RAGE, TLR2, and TLR4 to activate downstream signaling pathways and release inflammatory factors that lead to further damage^40^. Numerous studies demonstrated that the genetic or pharmacological inhibition of RAGE in animal models attenuated brain edema after injury and inhibited the pro-inflammatory response^41,42^. Thus, the present study examined whether RAGE was involved in the TSO IIA-mediated reduction of astrocyte swelling. The results of the study were in agreement with this hypothesis and demonstrated a role for RAGE in neuroinflammation and cell swelling, given our findings that RAGE inhibition alleviated increased nuclear NF-κB transport, IL-6 release, AQP4 accumulation on the plasma membrane, and astrocyte swelling after OGD/R. In addition, further exposure to exogenous rHMGB1 did not alter these effects. Further, the results showed that the efficacy of TSO IIA was similar to that of RAGE inhibitors. Taken together, the findings suggested that the regulation of HMGB1-mediated pro-inflammation by TSO IIA occurred through RAGE.

IL-6 is an important inflammatory cytokine mainly synthesized and secreted by astrocytes and microglia^43,44^. Laird et al. reported that HMGB1 triggered IL-6 release and IL-6 enhanced AQP4 expression and cellular swelling of brain astrocytes^29^. Neutralizing IL-6 abrogated the HMGB1-induced promotion of AQP4 expression in the plasma membrane and cell swelling^39^. Therefore, IL-6 seems to be one of the HMGB1-induced key inflammatory proteins to promote AQP4 expression and cell edema. In the present study, the increased IL-6 protein expression in the cytoplasm and extracellular medium after OGD/R was attenuated by treatment with TSO IIA. The co-treatment of astrocytes with TSO IIA and exogenous IL-6 abrogated the beneficial effects of TSO IIA on the overclustering of AQP4 in the plasma membrane, cell swelling, and LDH release. These results suggested that TSO IIA could alleviate the expression of the pro-inflammatory mediator, HMGB1, thereby abolishing the activation of the HMGB1/RAGE/NF-κB/IL-6 inflammatory axis. In turn, this interaction led to a decrease in the overexpression of AQP4 in the plasma membrane and astrocyte swelling after OGD/R.

Although this study had several strengths, it also had some inevitable limitations. The brain is a complex system with many different cell types, such as neurons, microglia, and endothelial cells, which interact with each other during the inflammation and edema process. This study only examined the effects of TSO IIA on astrocyte swelling. Further studies should be conducted in co-culture systems in vitro and in vivo to validate the findings of this study. Besides RAGE, other receptors of HMGB1, such as TLR2 and TLR4, and their downstream signaling factors are involved in the HMGB1-induced inflammatory response. These factors were not explored in this study and should be investigated in the future.

## Conclusions

The present study showed that the treatment with TSO IIA alleviated astrocyte swelling after OGD/R injury in vitro. It was suggested that this effect depended on the abrogation of the activation of the HMGB1/RAGE/NF-κB pathway, downregulation of the expression of the pro-inflammatory factor IL-6, and thus reduction of the excessive accumulation of AQP4 protein in the plasma membrane. Therefore, TSO IIA has the potential to be used as a new therapeutic option for brain edema after I/R-like injury.

## Materials and methods

### Animals and ethics approval

Two-day old Sprague–Dawley (SD) rat pups (licensed 21 CAE035; authorization no. 75-776) were supplied by the Laboratory Animal Center of Chongqing Medical University, China. All procedures were approved by the “Use Committee” and “Institutional Animal Care” of Chongqing Medical University, China, and followed the National Institutes of Health (NIH) guidelines (NIH Publication No. 80-23, revised 1996) on animal care and use. All efforts were made to minimize animal suffering, and pain and discomfort were carefully monitored during all the experiments. The animals underwent surgery randomly, and the outcome assessments were evaluated by investigators blinded to the experimental groups. This study is reported in accordance with ARRIVE guidelines (“Supplementary information”).

### Special reagents and antibodies

Special reagents and antibodies included recombinant HMGB1 (rHMGB1, ProSpec, Rehovot, Israel), IL-6 (PeproTech, NJ, USA), FPS-ZM1 (Sigma–Aldrich, MO, USA), anti-HMGB1 antibody (Abcam, Cambridge, UK), anti-AQP4 antibody (Abcam), anti-NF-κB antibody (Cell Signaling Technology, MA, USA), anti-IL-6 antibody (Millipore, MA, USA), anti-glyceraldehyde-3-phosphate dehydrogenase (GAPDH) antibody (Beyotime, Shanghai, China), anti-histone H3 antibody (Beyotime), and anti-Cadherin antibody (Beyotime). Tanshinone IIA was sourced from IMAM International Pharmaceuticals, Ltd. (Tianjin, China).

### Astrocyte isolation and culture

The astrocytes were prepared from primary cell cultures of neocortex tissue of cerebral hemispheres of SD rats as described in a previous study^45^. In short, 2-day-old SD rats were anesthetized with 5% isoflurane in 70% N_2_O/30% O_2_ and then decapitated to extract the brains. During the whole operation, the rectal temperature was maintained at 37 ℃ using a feedback-regulated water heating system. The brains were dissected and treated with 0.25% (*v*/*v*) trypsin solution for 10 min. The cells were cultured in a medium containing DMEM/F12 and 10% (*v*/*v*) fetal bovine serum in cell culture flasks with 95% air and 5% carbon dioxide, and the medium was changed twice a week. After 7–9 days of culture, the flasks with the cell cultures were shaken gently for 14 h to remove oligodendrocytes and microglia. After determining the levels of glial fibrillary acidic protein (GFAP; Santa Cruz, CA, USA) using immunofluorescence immunostaining, the culture comprised 95–99% of 15- to 20-day-old primary astrocytes^46^. Obtained astrocyte cultures were used respectively in subsequent experiments.

### Astrocyte model of OGD/R and treatments

OGD/R injury was used to establish an in vitro model of ischemia/reperfusion-like injury. Briefly, primary astrocytes were washed twice and cultured in serum-free DMEM without glucose at 37 ℃ in 5% CO_2_ and 95% N_2_ for 5 h (oxygen–glucose deprivation, OGD). Then, the cells were rinsed once and returned to the normal medium with 10% (*v*/*v*) fetal bovine serum and DMEM/F-12 in 95% air and 5% CO_2_ for 24 h (reoxygenation, R). The astrocyte cultures were then randomly assigned into seven different treatment groups.

1. TSO IIA group: The cells were treated with 1 μM/mL TSO IIA for 24 h before and throughout the OGD/R process.
2. TSO IIA + HMGB1 group: The cells were co-treated with TSO IIA as described in (1) and 1 µg/mL rHMGB1 during the reoxygenation phase.
3. FPS-ZM1 group: The cells were treated with 0.5 nM FPS-ZM1 for 24 h before and throughout the OGD/R process.
4. FPS-ZM1 + HMGB1 group: The cells were co-treated with FPS-ZM1 as in (3) and rHMGB1 as in (2).
5. TSO IIA + IL-6 group: The cells were co-treated with TSO IIA as in (1) and 1 ng/mL IL-6 during the reoxygenation stage.
6. OGD/R group: The cells were treated with the corresponding volume of vehicle solution for 24 h before and throughout the OGD/R process.
7. Control group: The cells were treated with the corresponding volume of vehicle solution without the OGD/R process.

The doses of TSO IIA, HMGB1, FPS-ZM1, and IL-6 were based on those found to be the most effective in preliminary trials (data not shown).

### Cell edema analysis

To estimate cell edema after OGD/R, the cell volumes were analyzed at different time points (1, 4, 12, and 24 h) during reoxygenation^47^. Briefly, 1 mM 3-O-methylglucose (3-OMG) and 0.5 μCi/mL [3H]-3-OMG (NEN, MA, USA) were added to the culture for 6 h. The radioactivity of the culture medium and the cell extract was then determined. The cell volume was expressed in mL/mg protein. A small portion of the cell extract was used for protein estimation using the BCA method as previously described.

### Measurement of cell morphology

The morphology of edematous astrocytes was observed by phase-contrast microscopy. After 4 h of reoxygenation, the cell edema was the most obvious, and the imaging of the cell morphology was carried out using a DMi8 Leica phase-contrast microscope.

### Transmission electron microscopy

After 4 h of reoxygenation, the cells were scraped into the culture medium from the plastic with a rubber policeman and centrifuged at 400*g* for 5 min to form a pellet. The supernatant was removed, and the pellet was fixed with 3 mM CaCl_2_, 2.5% glutaraldehyde, and 1% sucrose in 0.1 M sodium cacodylate buffer (pH 7.2) for 1 h at room temperature. After a buffer rinse, the samples were post-fixed with 1% osmium tetroxide in the buffer (2 h) on ice in the dark. The specimens were stained with 2% aqueous uranylacetate, dehydrated in a graded series of ethanol, and embedded in Eponate 12 resin. The samples were polymerized at 60 ℃ overnight. The ultrathin sections were prepared with a diamond knife on the Reichert-Jung Ultracut E ultramicrotome and picked up with naked 200-mesh copper grids. The grids were stained with 2% uranyl acetate in 50% methanol, and the cells were observed under a transmission electron microscope (No. 7600, Hitachi, Japan). Transmission electron microscopy was performed at the Johns Hopkins Institute for Basic Biomedical Sciences microscope facility.

### Measurement of lactate dehydrogenase activity

The cell culture medium was sampled after 1, 4, 12, and 24 h of reoxygenation to examine the cell damage by OGD/R. Lactate dehydrogenase (LDH) release rates in the medium were assessed using an LDH assay kit (Cat# C0016, Beyotime) following the kit manufacturer's instructions.

### Plasma membrane and nucleus isolation

The plasma membrane was isolated and collected as previously described to detect the expression of the AQP4 protein in the plasma membrane^48,49^. Briefly, the cells were washed with ice-cold Phosphate Buffer Saline (PBS) containing protease inhibitor cocktail, harvested, and centrifuged at 4000*g* (10 min). The pellets were frozen at − 80 °C for 2 h, subsequently homogenized in 0.2 mL of Tris–EDTA buffer (50 mM, pH 8) containing protease inhibitor cocktail, and centrifuged at 15,000*g* for 30 min. The particles were resuspended and centrifuged again at 15,000*g* for 30 min. The remaining particles were then dissolved in 100 mL of RIPA buffer, and proteins were extracted for further analysis.

The nuclear proteins were isolated and collected using a nuclear extraction reagent kit following the manufacturer's protocols (Cat# P0028, Beyotime) to detect the expression of NF-κB protein in the nucleus. Briefly, for every 20 µL of the cell precipitate, 200 µL of cytoplasmic protein extraction reagent A was mixed with Phenylmethanesulfonyl fluoride (PMSF) and vortexed. Then, 10 µL of the cytoplasmic protein extraction reagent B was added and centrifuged at 12,000*g* for 5 min. The remaining supernatant was completely absorbed. Further, 50 µL of PMSF-added nuclear protein extraction reagent was added to the precipitate and centrifuged at 4 °C and 12,000*g* for 10 min to obtain nucleoproteins.

### Western blot analysis

Western blot analysis was used to determine the protein levels of plasma membrane AQP4, cytoplasmic HMGB1, nuclear NF-κB, and cytoplasmic IL-6 at different time points during reoxygenation. The protein concentrations were determined using a BCA protein assay kit (Beyotime). Equal amounts of proteins were separated electrophoretically in 10% sodium dodecyl sulfate-polyacrylamide gel and transferred to polyvinylidene fluoride membranes (Merck Millipore, MA, USA) for immunoblot analysis. The blocked membranes were incubated with primary antibodies, washed, and then incubated with the appropriate IgG secondary antibodies. The primary antibodies used were as follows: AQP4 (1:250 dilution, Santa Cruz), HMGB1 (1:1000 dilution; Abcam), NF-κB (1:1000 dilution; Cell Signaling Technology), IL-6 (1:1000 dilution; Millipore), cadherin (1:1000, Beyotime), and histone H3 and GAPDH (1:1000, Beyotime) (25). The blots were cut prior to hybridisation with antibodies during blotting. The ECL system was used for detection, and densitometry was used for scanning membranes for semi-quantitative analysis. The expression levels of proteins in the plasma membrane, cytoplasm, and nuclear extracts were normalized to the levels of cadherin, GAPDH, and histone H3, respectively.

### Fluorescence immunostaining

Immunofluorescence double staining analysis was performed to examine the co-expression of GFAP and AQP4 proteins in astrocytes after 4 h of reoxygenation^50^. Briefly, the cells were fixed onto a coverglass with cold methanol. They were incubated with GFAP (1:500 dilution, Santa Cruz) and AQP4 (1:100 dilution, Santa Cruz) antibodies at 4 °C overnight. Subsequently, they were incubated with tetraethyl rhodamine isothiocyanate (TRITC)- or fluorescein isothiocyanate (FITC)-labeled IgG antibody at room temperature for 1 h. Normal rabbit serum instead of the primary antibody was used as a negative control. The coverslips were mounted on slides and examined using a confocal microscope (DMi8, Leica). Single-interference filter sets for green (FITC) and red (TRITC) filters were used. Calibrations were performed using the Ariol Scan application 4.0.1.5 (Leica Biosystems Richmond Inc.). After delineation of the scanning area (i.e., one entire ISET spot) at 5× magnification, the gain was set at a maximum (255) to eliminate the risk of fluorochrome bleaching. The exposure time was calibrated for each channel at 20× magnification. Using only one parameter (i.e., exposure time for adjusting fluorochrome exposure) allowed to compare settings between scans. All images were analyzed with the Ariol Review application 4.0.1.5 (Leica Biosystems Richmond Inc.).

### Enzyme-linked immunosorbent assay

The protein levels of HMGB1 and IL-6 released from astrocytes into the surrounding medium after OGD/R were measured using ELISA following the manufacturer's instructions (Beyotime). Briefly, the culture media were sampled, incubated for 1 h with antibodies targeting HMGB1 or IL-6, followed by incubation with an enzymatic working solution for 30 min at 37 ℃, and incubated with TMB solution for 15 min at 37 ℃ in the dark. Finally, the absorbance at 450 nm was measured to determine protein levels.

### Statistical analysis

Measurement data were expressed as mean ± error of the mean. Statistical comparisons were performed using one-way analysis of variance with Tukey’s post hoc test. Differences with a *P* value < 0.05 were considered statistically significant. Data were analyzed using SPSS 17.0.

## Supplementary Information

Supplementary Information.

## Data Availability

The datasets used and analyzed during the current study are available from the corresponding author on reasonable request.
