# Supplementary material for: Tanshinone IIA reduces AQP4 expression and astrocyte swelling after OGD/R by inhibiting the HMGB1/RAGE/NF-κB/IL-6 pro-inflammatory axis
Source: Sci Rep. 2022 Aug 18;12:14110. doi: 10.1038/s41598-022-17491-7 (PMC9388613; doi:10.1038/s41598-022-17491-7)
Supplement: Supplementary file 1 — Supplementary Information. [file 41598_2022_17491_MOESM1_ESM.pdf]

We had performed our samples for each antibody in a full-length, original, unprocessed blot to confirms specific detection of the target antigen.
The blots blow were cut prior to hybridisation with antibodies during blotting.

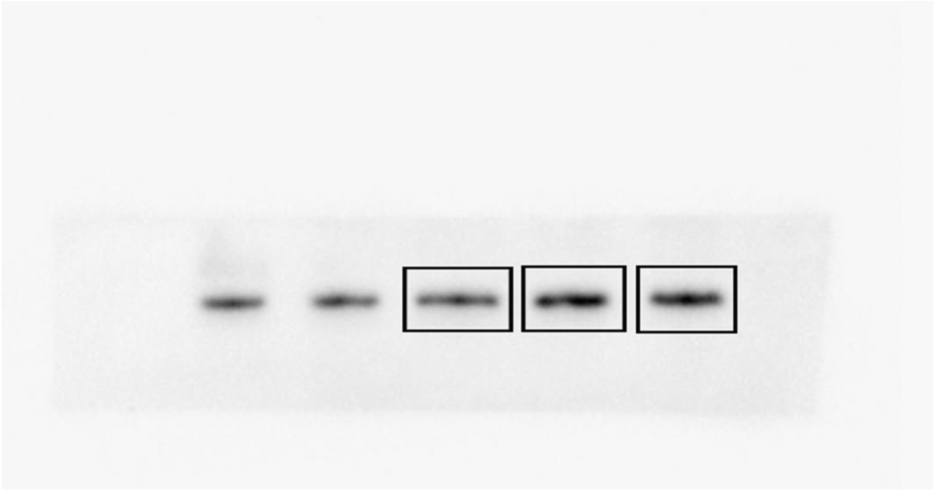

↑ Fig.2A-AQP4 (34 kDa)

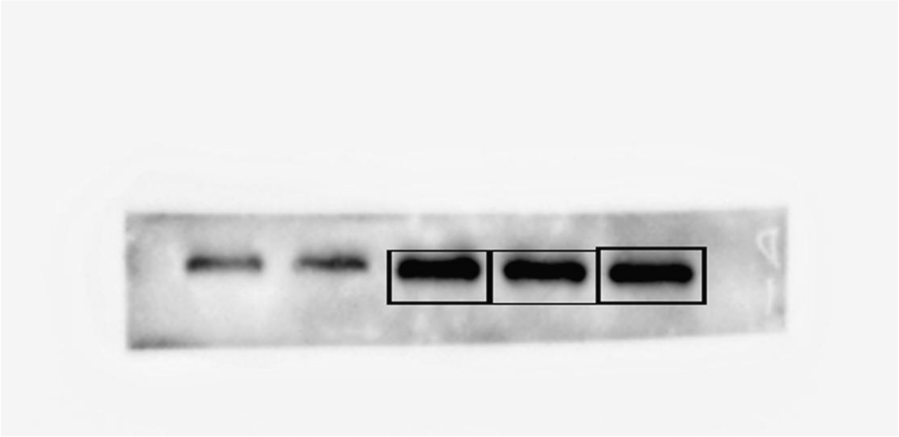

↑ Fig.2A-Cadherin (140 kDa)

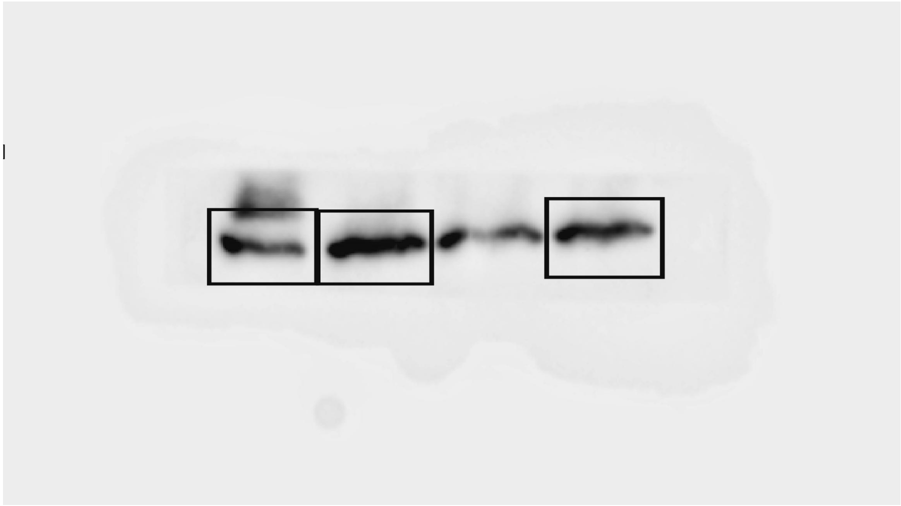

↑ Fig.3A-HMBG1 (25 kDa)

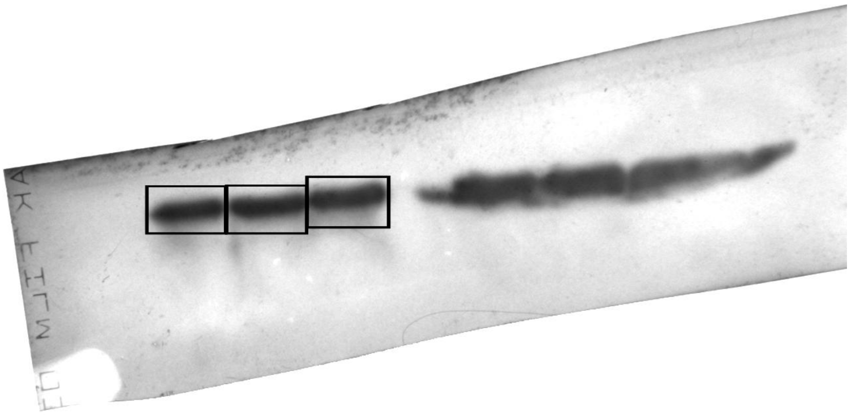

↑ Fig.3A-GAPDH (36 kDa)

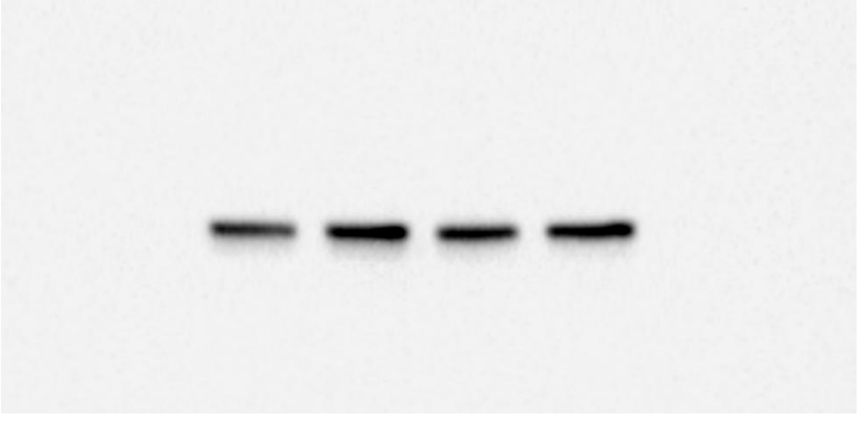

↑ Fig.3C-NF-kB (65kDa)

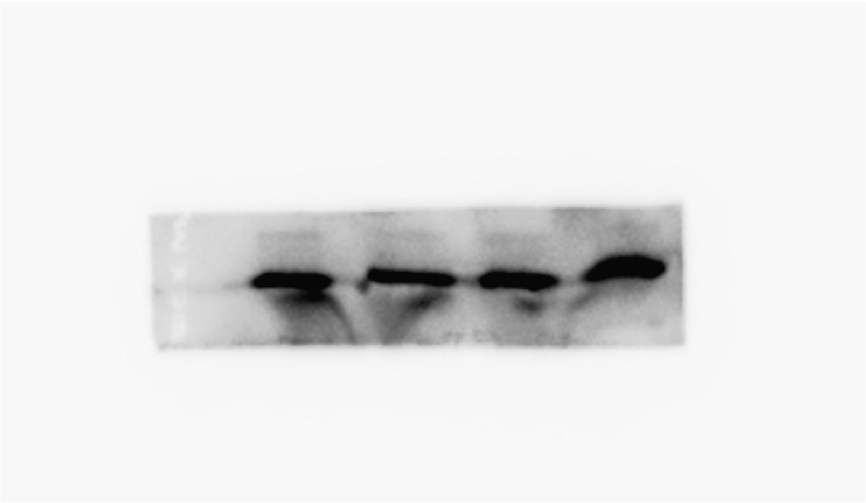

↑ Fig.3C-Histone (16 kDa)

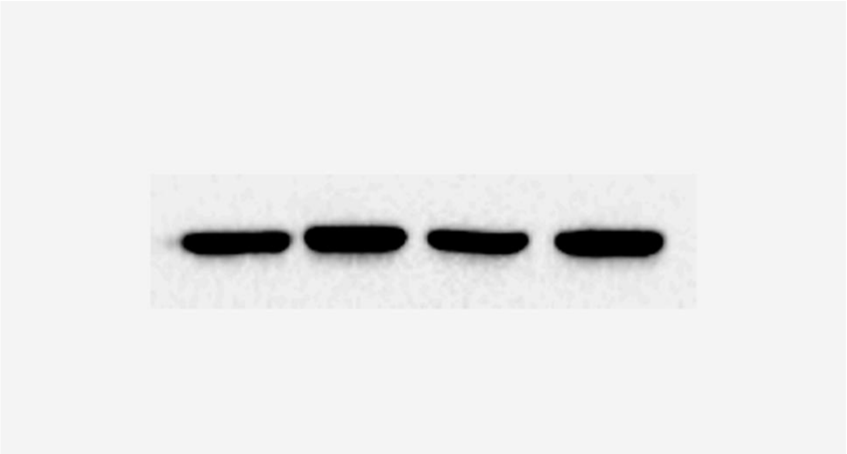

↑ Fig.3D-IL-6 (24 kDa)

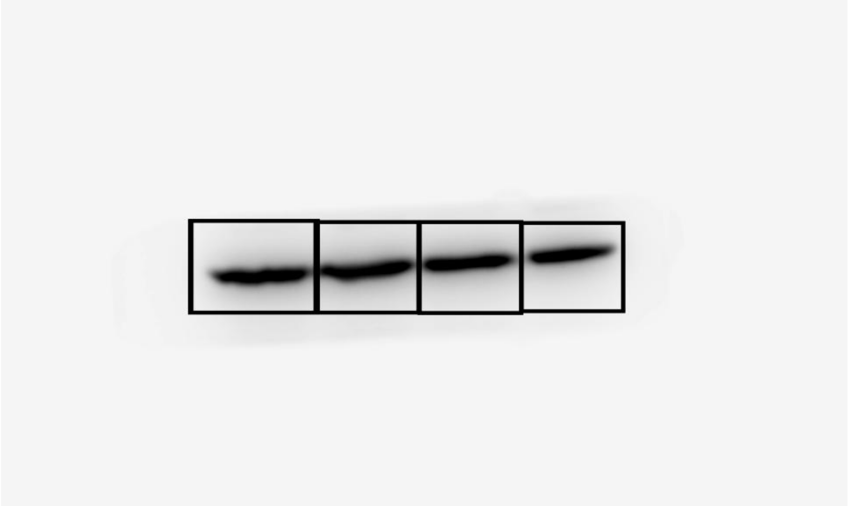

↑ Fig.3D-GAPDH (36 kDa)

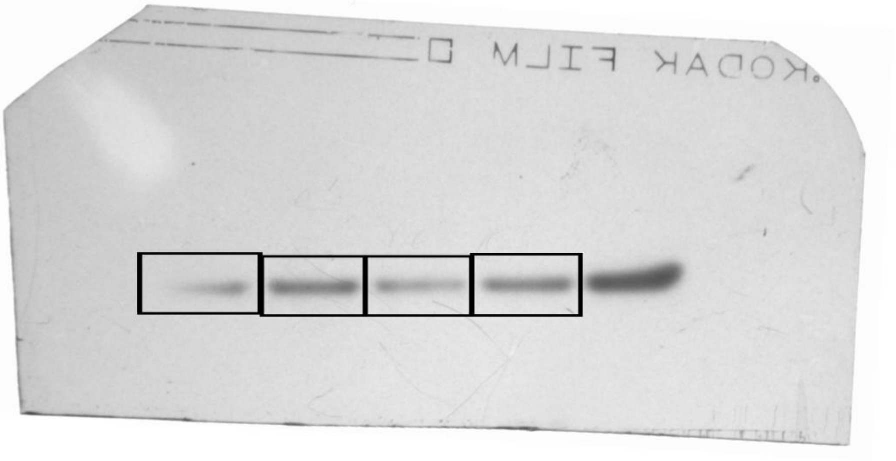

↑ Fig.3F-AQP4 (34 kDa)

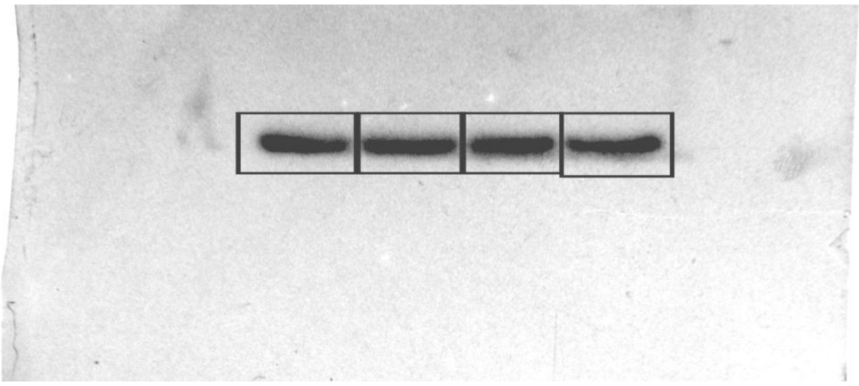

↑ Fig.3F-Cadherin (140 kDa)

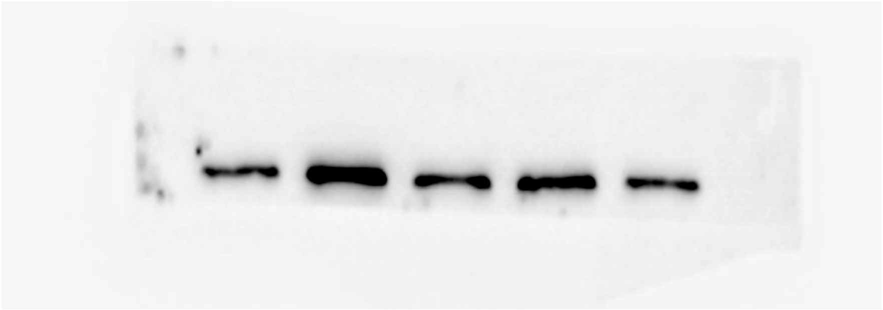

↑ Fig.4A-NF-k**B (**65kDa**)**

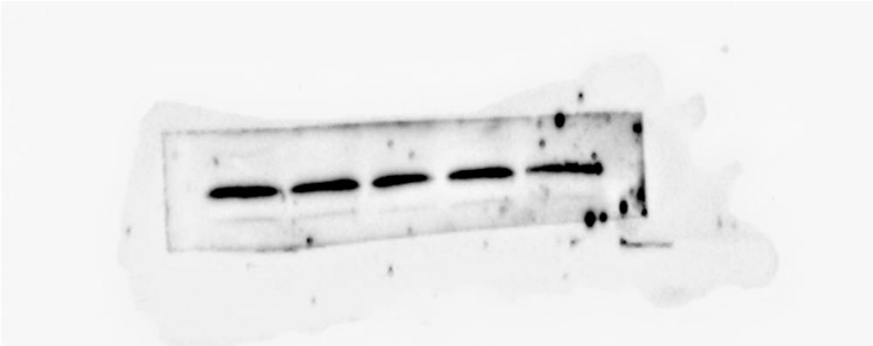

↑ Fig.4A Histone H3 (16 kDa)

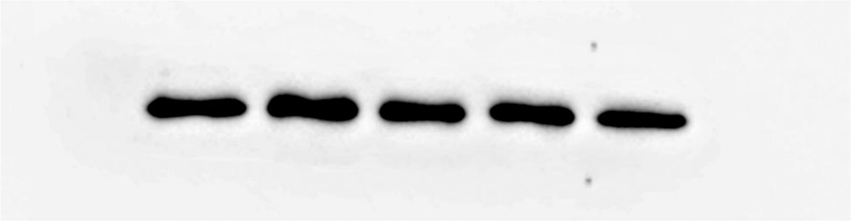

↑ Fig.4B-IL6 (24 kDa)

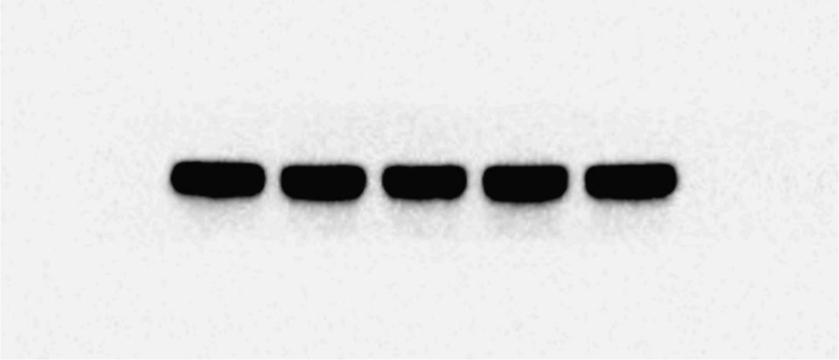

↑ Fig.4B-GAPDH (36 kDa)

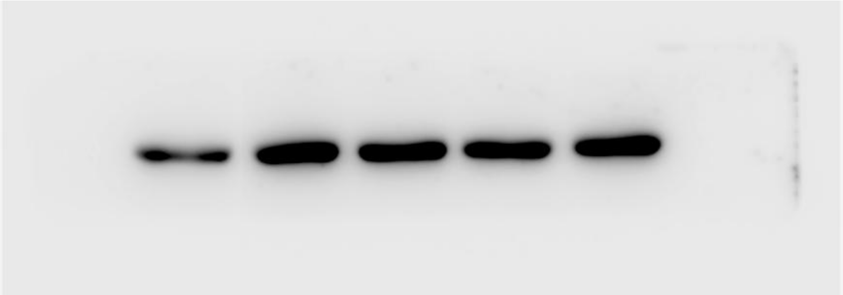

↑ Fig.4D-AQP4 (34 kDa)

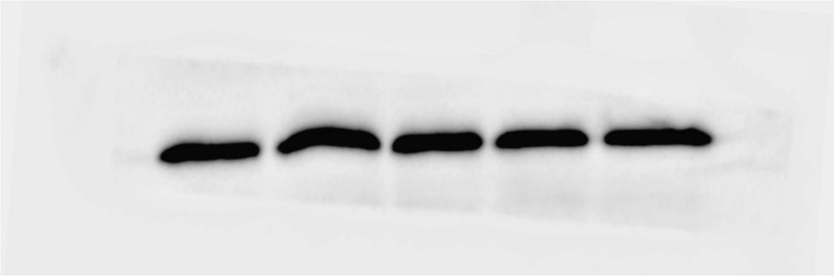

↑ Fig.4D-Cadherin (140 kDa)

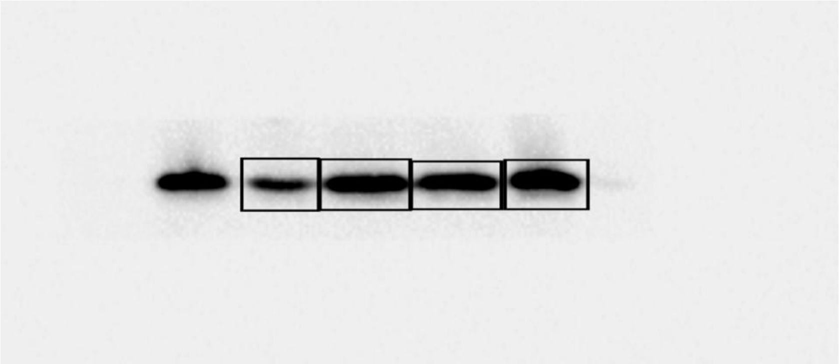

↑ Fig.5A-AQP4 (34 kDa)

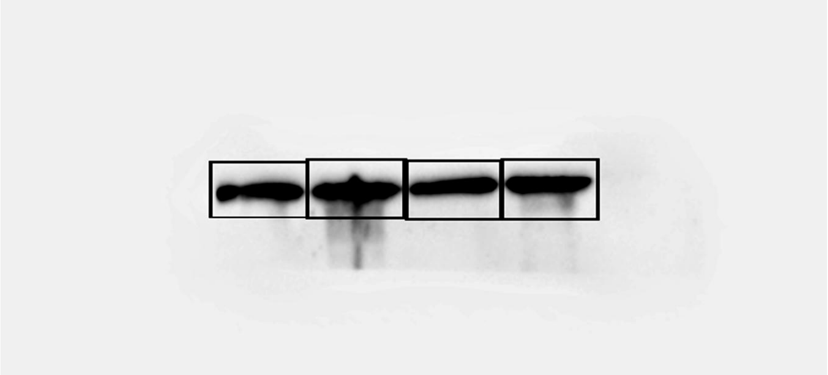

↑ Fig.5A-Cadherin (140 kDa)
